# Codon Usage and Context Analysis of Genes Modulated during SARS-CoV-2 Infection and Dental Inflammation

**DOI:** 10.3390/vaccines10111874

**Published:** 2022-11-06

**Authors:** Rekha Khandia, Megha Katare Pandey, Azmat Ali Khan, Igor Vladimirovich Rzhepakovsky, Pankaj Gurjar, Mohmed Isaqali Karobari

**Affiliations:** 1 Department of Biochemistry and Genetics, Barkatullah University, Bhopal 462026, India; 2 Department of Translational Medicine, All India Institute of Medical Sciences, Bhopal 462020, India; 3 Pharmaceutical Biotechnology Laboratory, Department of Pharmaceutical Chemistry, College of Pharmacy, King Saud University, Riyadh 11451, Saudi Arabia; 4 Medical and Biological Faculty, North Caucasus Federal University, 355009 Stavropol, Russia; 5 Department of Science and Engineering, Novel Global Community Educational Foundation, Hebersham, NSW 2770, Australia; 6 Conservative Dentistry Unit, School of Dental Sciences, Universiti Sains Malaysia, Kubang Kerian 16150, Malaysia; 7 Department of Conservative Dentistry & Endodontics, Saveetha Dental College & Hospitals, Saveetha Institute of Medical and Technical Sciences University, Chennai 600077, India; 8 Department of Restorative Dentistry & Endodontics, University of Puthisastra, Phnom Penh 12211, Cambodia

**Keywords:** *BMAL1*, *ACE2*, *TMPRSS2*, *FURIN*, *CD147*, dental, periodontitis, SARS-CoV-2, codon context, rare codons

## Abstract

The overexpression of SARS-CoV-2 primary receptors and co-receptors (*ACE2*, *TMPRSS2*, *FURIN*, and *CD147*) enhance the likeliness of SARS-CoV-2 infection. The genes for same receptors are overexpressed in the periodontal tissues of periodontitis patients. On the other hand, *BMAL1* is recognized to play a crucial role in regulating pulmonary inflammation and enhancing susceptibility to viral infection. Silenced *BMAL1* disrupts circadian transcriptional regulations, enhances vulnerability to SARS-CoV-2 infections, and may trigger the further production of TNF-α and other pro-inflammatory cytokines that propagate the cytokine storm and exacerbate periodontal inflammation. Therefore *ACE2*, *TMPRSS2*, *FURIN*, *CD147*, and *BMAL1* are the crossroads between SARS-CoV-2 and Periodontitis genes. The enhanced expression of *ACE2*, *TMPRSS2*, *FURIN*, and *CD147* and the diminished expression of *BMAL1* may be a strategy to check both ailments simultaneously. In gene manipulation techniques, oligos are introduced, which contain all the necessary information to manipulate gene expression. The data are derived from the studies on genes’ molecular patterns, including nucleotide composition, dinucleotide patterns, relative synonymous codon usage, codon usage bias, codon context, and rare and abundant codons. Such information may be used to manipulate the overexpression and underexpression of the genes at the time of SARS-CoV-2 infection and periodontitis to mitigate both ailments simultaneously; it can be explored to uncover possible future treatments.

## 1. Introduction

COVID-19 was caused by the SARS-CoV-2 virus and had a catastrophic effect on the world’s demographics leading to more than 6 million deaths worldwide up to early 2022 and is emerging as the most critical health crisis. The symptoms of COVID-19 include fever, malaise, dry cough, sore throat, dyspnea, and respiratory complications that can eventually lead to multiple health problems and death [1]. Nasal and oral cavities play a significant role in preventing some pathogens. COVID-19 patients have a higher viral load in saliva and nasopharyngeal secretions [2], and long-term SARS-CoV-2 RNA shedding has also been reported [3]. The presence of the virus in saliva and nasopharyngeal secretions indicates the possibility of the virus spreading through the oral and nasal routes. For entry into host cells, the SARS-CoV-2 virus uses the ACE2 receptor [4] and spike protein priming through serine protease *TMPRSS2* [5]. *TMPRSS2* is also required for viral spread and pathogenesis [6]. Another protein that helps in spike priming is FURIN, and priming is an essential step of virus binding to the ACE2 receptor. During the biosynthesis of the virus, the S protein is cleaved by FURIN protease into S1 and S2 units which remain associated [7]. S1 subunits bind to the receptor, and S2 anchors the virion, mediating fusion between the virus and the host cells [8]. There are emerging reports pointing towards the role of CD147 as a receptor for SARS-CoV-2 [9]. Therefore *ACE2*, *TMPRSS2*, *CD147*, and *FURIN* may be considered receptors for SARS-CoV-2 entry [10,11]. It is obvious that if the protein expression level of receptors for SARS-CoV-2 entry is modulated, it will also affect the severity of infection. Experimentally the notion is supported by the work of Dobrindt and colleagues [12], who reported that host gene manipulation using the shRNA strategy to knockdown the expression of *ACE2*, *CD147*, *FURIN*, and *TMPRSS2* in neurons of SARS-CoV-2-infected cells resulted in the reduction in viral RNA expression to 26.6%, 61.1%, 37.7%, and 38.7% for *ACE2*, *CD147*, *FURIN*, and *TMPRSS2* genes, respectively [12]. In various bacterial and viral infection models, the loss of the circadian gene *BMAL1* resulted in increased viral burden and disease severity [13]. 

*ACE2*, *TMPRSS2*, *FURIN*, and *CD147*, which are SARS-CoV-2 primary receptors and co-receptors, are overexpressed in the periodontal tissues of periodontitis patients, presenting with inflammation, periodontal pathogens, and damage-induced pyroptosis [14]. Immunohistochemistry results also supported that SARS-CoV-2 invasion-related molecules are abundant in the oral cavity. Based on the National Electronic Health Records of the State of Qatar between February and July 2020, an association between periodontitis and severe COVID-19 complications, such as death, ICU admissions, and/or assisted ventilation, has been observed [15], and periodontitis can be a risk factor for COVID-19 severity. 

The *BMAL1* gene regulates the innate immune system, and its disruption mediates the release of pro-inflammatory cytokines, which are involved in periodontitis, an inflammatory disorder [16]. Diminished BMAL1 may trigger TNF-α and other pro-inflammatory cytokines production leading to cytokine storm and exacerbating periodontal inflammation [17]. 

Other than *ACE2*, *BMAL1*, *CD147*, *FURIN*, and *TMPRSS2*, more genes, including *CD26(DPP4)*, *IFITM3*, *HLA*, *ABO*, *SLC6A20*, *GSTT1-M1*, and *DBP* [18], have also been implicated in SARS-CoV-2 and periodontitis. *CD26* has a role in expressing the essential regulatory genes participating in SARS-CoV-2 internalization [19]; however, at periodontitis location, the expression of *CD26* is not significantly altered [20]. Periodontitis induced an 8-fold higher expression of the *IFITM3* gene [21], while the artificial overexpression of *IFITMs* blocks SARS-CoV-2 infection [22]. Among the ABO blood group system, a higher percentage of “O” group patients were found in the periodontitis group, while group “O” of the same presented a lower risk of SARS-CoV-2 infection leading to severe disease [23]. The aggressive forms were statistically associated with the double null GSTM1 [24]. 

Vitamin D binding protein (DBP) showed a positive correlation (*p* < 0.05) between COVID-19 prevalence, mortality rates, and GT genotype among all populations. On the other hand, it showed a significant negative correlation (*p* < 0.05) between prevalence, mortality rates, and TT genotype at the rs7041 locus among all populations [25]. Significantly high levels (*p* < 0.05) of serum DBP were observed in periodontitis patients, with a significant linear relationship (*p* = 0.005) between the levels of DBP and the severity of periodontitis [26]. Contrarily low plasma DBP has been documented in patients with acute respiratory distress syndrome (ARDS) associated with SARS-CoV-2 infection. Since the frequency of *GSTM1*^−/−^, *GSTT1*^−/−^, and *GSTM1*^−/−^/*GSTT1*^−/−^ is higher in severe COVID-19 patients [27] and the aggressive forms of periodontitis were associated with the double null *GSTM1* and *GSTT1* combination [24]. 

In attempts at gene manipulation, gene therapy has been employed. To enhance the gene expression, a codon-optimized copy of the gene may be inserted into the engineered virus as a delivery vector where the engineered virus contains a minimal amount of viral DNA, just sufficient for genetic material packing [28]. Adenovirus, adeno-associated virus, lentivirus, and herpesvirus are a few popular viral vectors. For example, in Parkinson’s patients, dopamine levels are decreased, and by increasing the L-DOPA levels, dopamine levels may be augmented. For augmentation, an aromatic L-amino acid decarboxylase (*AADC*) gene is delivered that converts L-DOPA to dopamine [29]. To suppress gene expression, siRNA or antisense RNAs are used [30]. Information, such as nucleotide composition, dinucleotide patterns, relative synonymous codon usage, codon usage bias, codon context, and rare and abundant codons, may be utilized in constructing the oligos that may further be used in gene therapy techniques.

## 2. Materials and Methods

### 2.1. Sequence Retrieval

The transcripts for each gene sequence were retrieved from National Center for Biotechnology Information. For each gene, the transcripts resulting in a protein product were studied. All the sequences had a base number in multiples of three and did not contain stop codons in between the sequence. In the present study, 06, 51, 06, 07, and 03 transcripts qualified for *ACE2*, *BMAL1*, *CD147*, *FURIN*, and *TMPRSS2* genes, respectively (accession ID, nucleotide composition, and RSCU (defined in Section 2.4) are given in Supplementary Materials).

### 2.2. Compositional Analysis

Base composition determines many of the molecular features of any genome, including codon usage [31,32]. Subsequently, it influences other factors, such as the fine-tuning of gene expression [33], protein translation efficacy [34], the tuning of the protein co-translational folding process [35], stress regulation during the starvation condition [36] or oscillations in gene expression during the cell cycle [37]. The overall percent composition of nucleotides A, T, C, and G at the third codon position was determined (%A3, %T3, %C3, %G3, and %GC3).

### 2.3. Dinucleotide Odds Ratio Analysis

Dinucleotide bias affects the overall codon usage bias in several organisms. Moreover, their differential representation is observed in viruses. The expected occurrence of dinucleotide is called the odds ratio, and a value above 1.23 and below 0.78 is conserved as overrepresented and underrepresented dinucleotides, respectively [38]. The odds ratio was calculated using the formula:

$$Pxy=\frac{fxy}{fxfy}$$

where *fx*, *fy*, and *fxy* represent the frequency of nucleotide *x*, *y*, and dinucleotide *xy*, respectively.

### 2.4. Relative Synonymous Codon Usage (RSCU)

Except for Trp and Met and stop codons, all other codons are encoded by two or more than two codons. RSCU refers to the ratio of the observed frequency to the expected frequency of a specific codon out of many synonymous codons present for a single amino acid and is used to standardize the codon usage bias between different sequences [39]. An RSCU value of 1 indicates no bias in codon usage and all codons are used equally. An RSCU value of less than 0.6 and above 1.6 represents underrepresentation and overrepresentation, respectively. The RSCU value of each codon was calculated using the program CodonW 1.4.2.

### 2.5. Rare Codon Analysis

Rare codon ramps are often used as a rate-limiting steps for translating protein so that the translated protein may be folded appropriately [40]. Moreover, recoding viral genes using rare codons leads to virus attenuation [41]. The number of rare codons was calculated using the Anaconda 2.0 software [42]. Furthermore, the number of rare codons was normalized for all the transcripts.

### 2.6. Codon Context Analysis

Codon pair context is the possibility of the occurrence of two codons together. Few codon pairs are reported to be greater in number than other codon pairs. The frequency tables were prepared through Anconda2.0 software, which permits the contingency table’s statistical analysis and indicates whether the context is significantly biased. The chi-square (χ^2^) test of independence identifies the preferred and rejected pairs of codons in any transcript [43]. 

## 3. Results

### 3.1. Compositional Analysis

**Figure 1:**
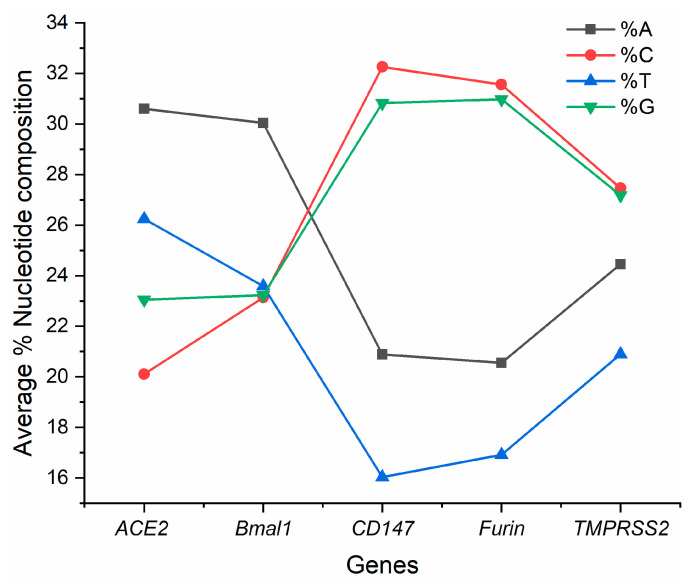
Average per cent composition of the genes.

The composition of all five genes is given in Figure 1. Maximum variation within transcripts was observed for the *CD147* gene. However, in the *BMAL1* gene, despite having the maximum number of transcripts, the variation in nucleotide composition was significantly less. The analysis revealed that %T and %A are low in *CD147, FURIN*, and *TMPRSS2* genes while high in *ACE2* and *BMAL1*, and the opposite is true for %C and %G.

### 3.2. Odds Ratio Revealed Two Distinct Odds Ratio Pattern Sets

Odds ratios show the underrepresentation or overrepresentation of certain dinucleotides. For example, in Human Rhinoviruses A, B, and C, significantly different (*p* < 0.001; Dunns Repeated Measures test) occurrence of CpG, CpA, and TpG dinucleotides has been observed along with a small borderline underrepresentation of TpA and CpG. The composition also influences the odds ratio for a dinucleotide pair, and Megremis and colleagues [44] found the occurrence of TpA dinucleotide in an inversely correlated manner with overall %CG and %TA composition (*p* < 0.0001).

**Figure 2:**
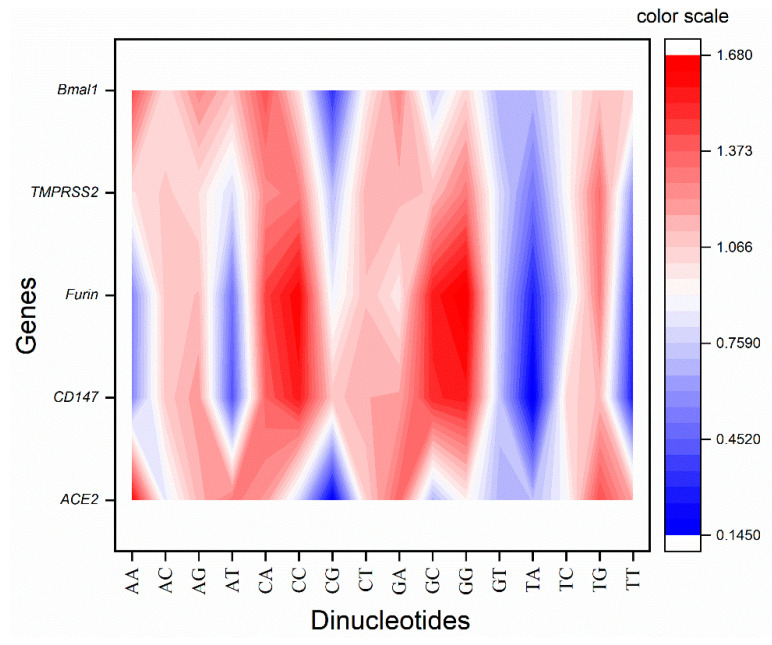
Contour plot for the dinucleotide odds ratio. The blue color shows underrepresentation, while the red shows an overrepresentation of dinucleotides.

In the present study, five genes were envisaged where *BMAL1* is found to be downregulated, and *ACE2*, *CD147*, *FURIN*, and *TMPRSS1* are upregulated in periodontitis conditions and the SARS-CoV-2 infection. CpT dinucleotide is present nearly unbiasedly in the transcripts of all the genes. *CD147* and *FURIN* genes showed an almost similar pattern of dinucleotide preference for ApA, ApC, ApG, ApT, CpA, CpC, CpT, GpC, and GpG dinucleotides. On the other hand, *ACE2* and *BMAL1* showed similar dinucleotide patterns in dinucleotide ApA, ApC, CpC, CpG, CpT, GpA, GpC, and GpG. CpA, CpC, and GpG are overrepresented, while TpT dinucleotide is underrepresented in the *CD147*, *FURIN* and *TMPRSS2* genes’ set. TpA is underrepresented in all the genes envisaged. Similarly, GpT is also underrepresented in all the genes but TMPRSS2. Based on the odds ratio, the envisaged genes may be divided into two groups, one comprising *CD147*, *FURIN* and *TMPRSS2* and the other comprising *ACE2* and *BMAL1* (Figure 2).

### 3.3. RSCU Analysis Revealed G/C Ending Codons Preference in CD147, FURIN, and TMPRSS2 Gene Set While A/T Ending Codons in ACE2 and BMAL1 

**Figure 3:**
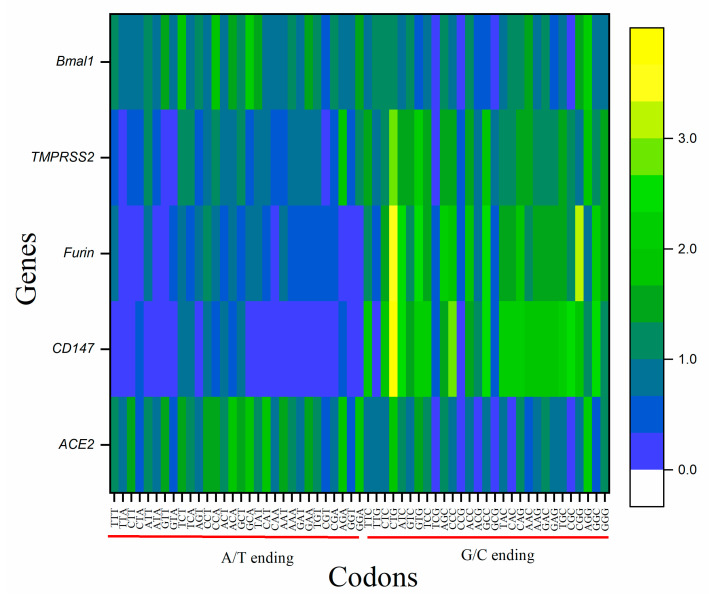
Heat map analysis for RSCU values of A/T and G/C ending codons of *ACE2*, *CD147*, *FURIN*, *TMPRSS2*, and *BMAL1*. Analysis revealed the dominance of G/C ending codons in *CD147*, *FURIN*, and *TMPRSS2* genes while the dominance of A/T ending codons in *ACE2* and *BMAL1*.

In the studied five genes, two patterns in RSCU were observed. Genes *CD147*, and *FURIN*, and *TMPRSS2* showed the similar overrepresentation of C/G ending codons, while *BMAL1* and *ACE2* genes set showed a similar pattern and a preference for A/T ending codons. Three codons, TCG, CCG, and GCG, were underrepresented in all the genes, while CTG and GTG codons were overrepresented in all the genes but Bmal (Figure 3). *CD147* was the gene that bypassed eight codons and did not use TTA, GTT, TGT, CGT, CGA, TAT, CAT, and CAA even for a single time in the transcripts. The *ACE2* gene bypassed TCG, CCG, and GCG codons. *BMAL1*, *FURIN*, and *TMPRSS2* genes used all available codons. The RSCU values of individual genes corresponding to their amino acid are given in Table 1.

### 3.4. Codons CGT, TCG and CTA Are Rarely Used in All the Genes

**Figure 4:**
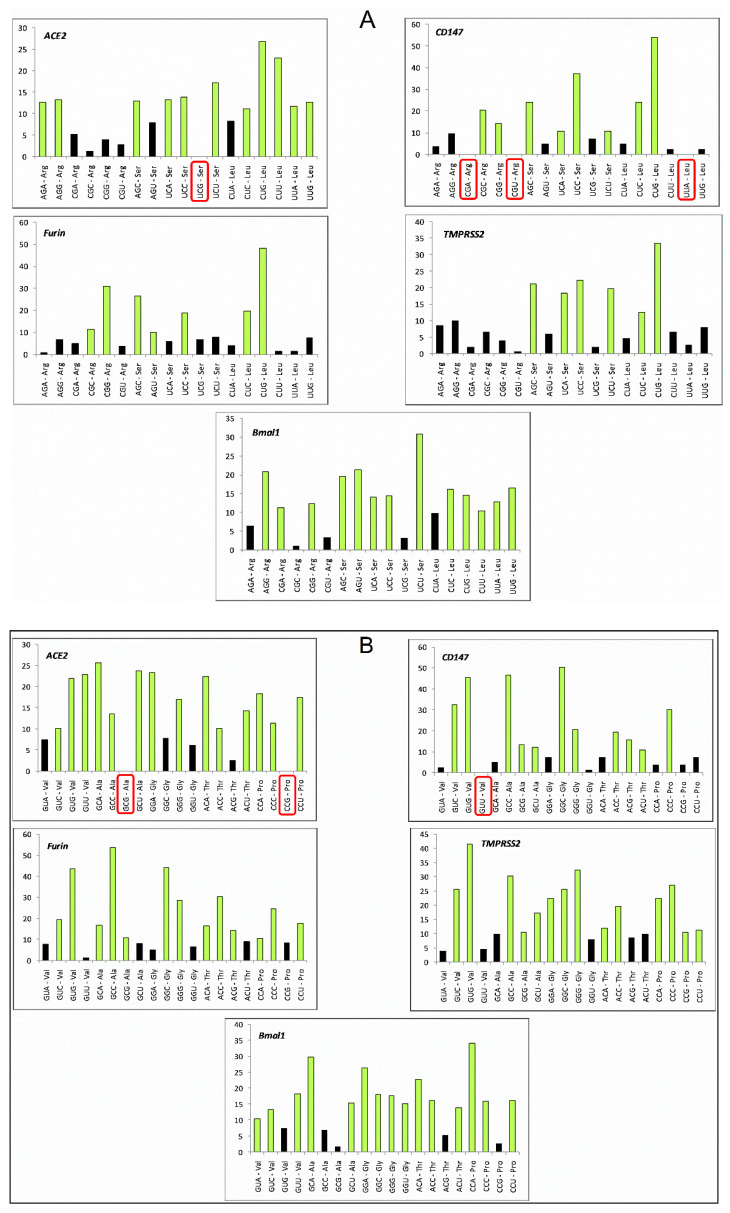

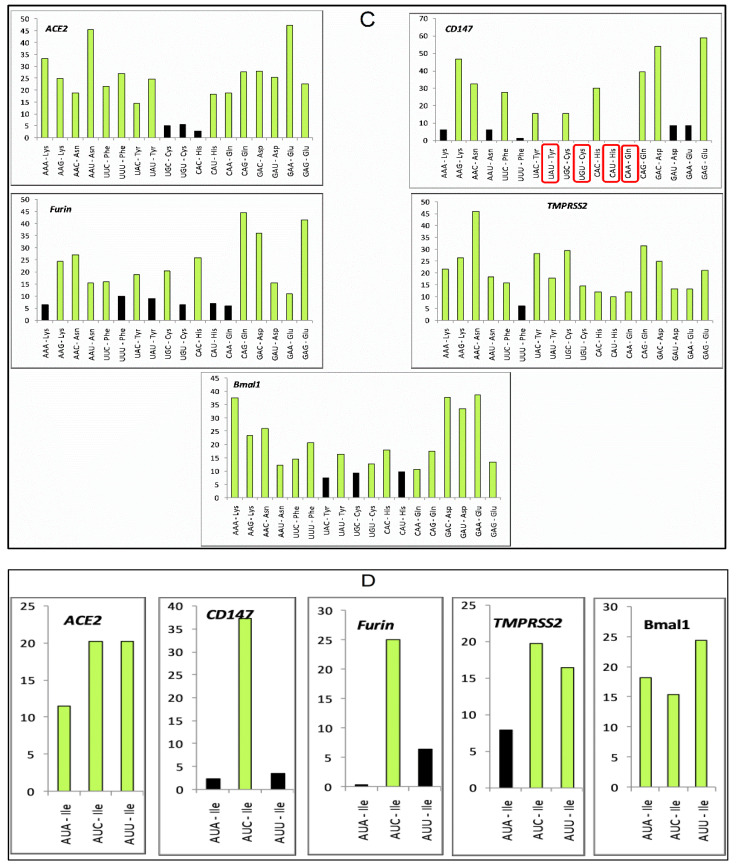
Rare codons in (**A**) Six-fold, (**B**) four-fold, (**C**) two-fold, and (**D**) three-fold degenerate codons in *ACE2*, *BMAL1*, *TMPRSS2*, *FURIN*, and *CD147* genes. Codons less than 10 in number in a standard transcript of 1000 bp (normalized transcript length) were considered rare. Codons that are altogether absent in the transcript of a gene are boxed with red color.

Codon usage bias is ruled by several mechanisms, resulting in various consequences. Optimal and unbiased codons are used in mRNA to be robustly translated [44]. On the other hand, the abundance of rare codons results in slower translation rates and deleteriously affects gene expression [45] and, in a few instances, reduces translational accuracy [46]. In the case of SARS-CoV-2, codon bias influences the host–pathogen interaction too. The presence of a specific rare codon is position-specific, and the presence at the 5′ end increases translational efficiency [47]. In the present study, we investigated six-fold, four-fold, two-fold, and three-fold degenerate codons separately (Figure 4). Among six-fold degenerated codons, CGT, TCG, and CTA encoding for Arg, Ser, and Leu, respectively, are rarely used (frequency less than 10 in 1000 bp) in all the five genes envisaged that are involved both in the periodontitis and the SARS-CoV-2 infection. Furthermore, AGA and CGA are the codons that were rare in four genes (codon AGA is not rare in *ACE2* and CGA is not rare in *BMAL1*). For four-fold degenerate codons, GTA (Val), GGT (Gly), and CCG (Pro) are rare in four out of five genes (GTA, GGT, and CCG are not rare in *BMAL1* and *TMPRSS2* genes, respectively).

### 3.5. Codon Context Analysis Revealed Val (GTG) Initiated Codon Pair Abundance in CD147, FURIN, and TMPRSS2

There is the presence of a specific bias for a certain combinations of codons [48]. The impact of codon context is further strengthened by pieces of evidence produced by codon pair deoptimization [49,50] and codon pair optimization [51]. Table 2 presents the top 30 codon pairs in the genes.

The analysis revealed that CAA-GAA, GTG-CTG, GTG-GCC, GTG-TGC, and GAA-TAT are the most abundantly found codon pair in *ACE2*, *CD147*, *FURIN*, *TMPRSS2*, and *BMAL1* genes, respectively. In *CD147*, *FURIN*, and *TMPRSS2* genes Val (GTG)-initiated codon pairs were most abundant, while in *ACE2* and *BMAL1* genes, Gln (CAA)- and Glu (GAA)-initiated codons were most abundant. Among all genes, *TMPRSS2* showed preference for Asn (AAC-CCC, AAC-AAU)-, Phe (UUG-AAC, UUC-AUG)-, Tyr (UAU-GAC, UAC-GGG, UAC-CAA)-, Val (GUC, GUG)-, Gly (GUG-UAC, GUC-GAU)-, Gly (GGG-GCC, GGA-UAC)-, Ala (GCG-CUG, GCC-UGC, GCC-GGC)-, Asp (GAC-UGG, GAC-UCC)-, Glu (GAA-AAC, GAA-AAA)-, Pro (CCU-CUG, CCC-ACU)-, and Gln (CAG-UAC, CAG-CCC)-initiated codons. *CD147* gene showed 25 codon pairs (GAG-GAC, GGC-UCC, CUG-AAG, UCA-GAG, GUG-AAG, GCG-CUG, GCC-CUC, CUG-GUC, CCC-GGC, CCC-GAG, AUC-AUC, ACU-GAC, ACG-GCC, AAC-GGC, UUC-GUG, UCC-GAC, UCC-AAG, GGC-CAG, GGC-ACC, GCC-GGC, GAC-GAC, GAC-CAG, CUG-GGC, AAU-GAC) to be highly used, followed by *FURIN* (three codon pairs, GUG-GCC, GCC-AAC, CUG-GGC) and *BMAL1* (two codon pairs, GAA-UAU, AAA-GAU).

**Figure 5:**
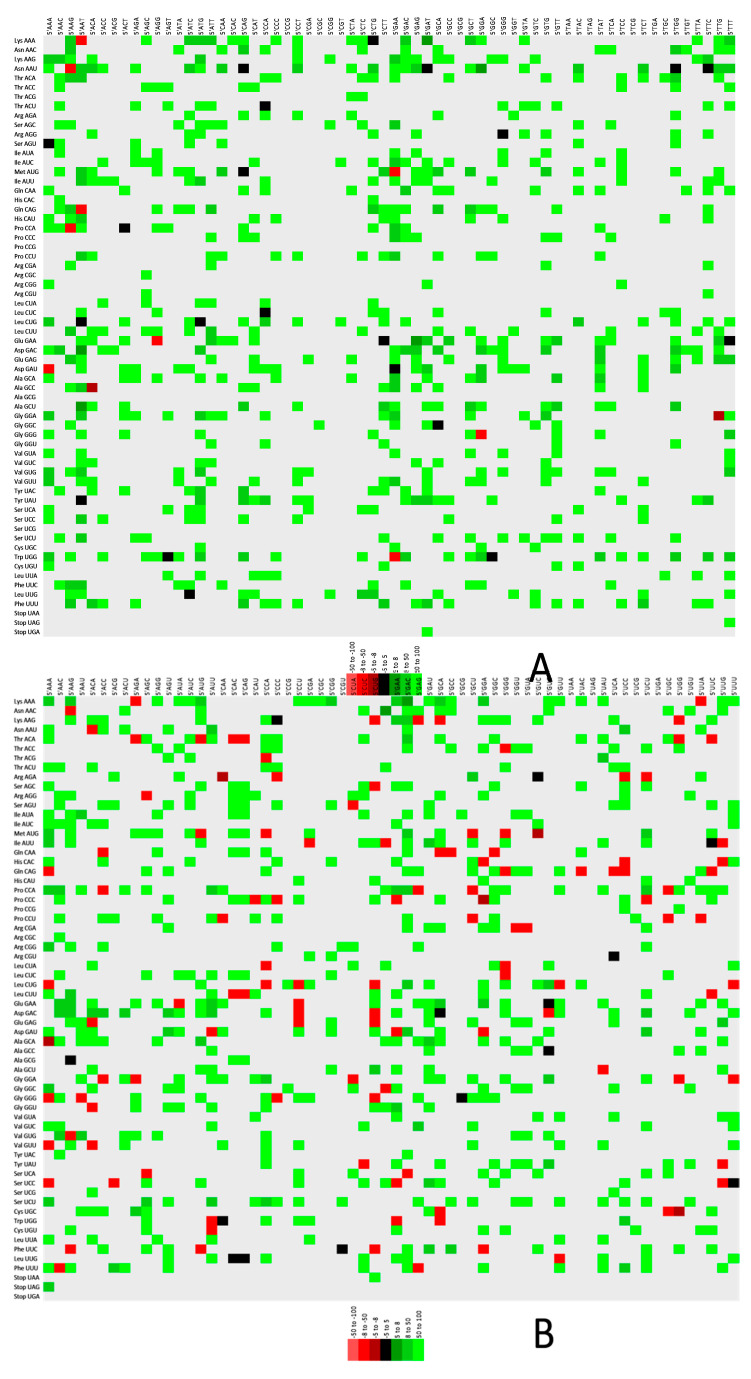

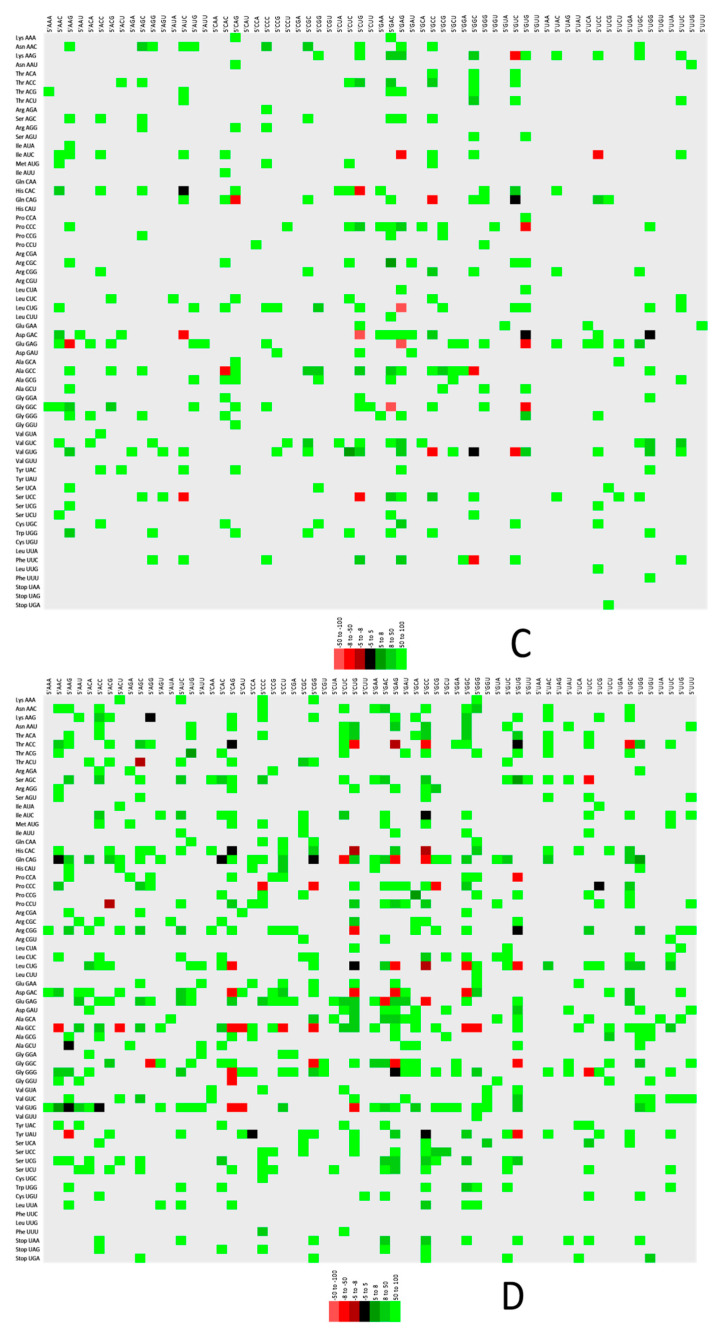

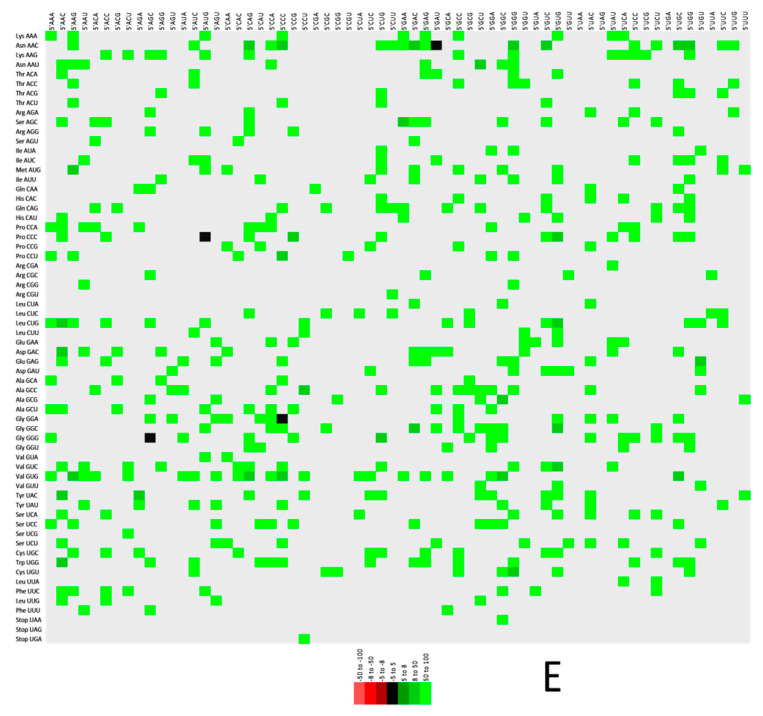
Codon context analysis for (**A**) *ACE2*, (**B**) *BMAL1*, (**C**) *CD147*, (**D**) *FURIN*, and (**E**) *TMPRSS2* genes. The green color shows the preferred codon pair, while the red color represents the rejected codon pairs. Statistically insignificant values are expressed as black. The grey color indicates unrepresented codon pairs.

Figure 5A–E demonstrates the codon context in two color-coded maps. Strongly preferred codon context bias is depicted as green, while firmly rejected codon context is shown as red. In the case where the 3′ context is not strongly biased, it is defined as black, revealing that in *TMPRSS2* gene, the rejected codon pairs are absent.

## 4. Discussion

COVID-19, caused by the SARS-CoV-2 virus, emerged as a severe health problem leading to the death of more than 6 million worldwide up to early 2022 [1]. *ACE2*, *TMPRSS2*, *CD147*, and *FURIN* act as receptors and coreceptors for SARS-CoV-2 entry. Their overexpression led to the enhanced severity and knocking down the expression of these genes by shRNA strategy, showed a reduction in viral protein expression [12]. Similar genes are overexpressed in the periodontal tissues of periodontitis patients [14]. Contrary to the four genes mentioned above, the loss of circadian gene *BMAL1* shows enhanced viral burden and disease severity [13]. Diminished *BMAL1* may trigger TNF-α and other pro-inflammatory cytokines production leading to cytokine storm and exacerbating periodontal inflammation [17]. 

Therefore, based on the above evidence, we studied five genes at the crossroads of periodontitis and SARS-CoV-2. Gene manipulations that alter the gene expression level of these genes might be helpful in simultaneously controlling both the SARS-CoV-2 infection and periodontitis. Here the upregulation of *ACE2*, *TMPRSS2*, *FURIN*, *CD147*, and downregulation of *BMAL1* genes predispose towards the ailment. Therefore, if a strategy can be planned to modulate the gene expression of these genes in the future, it might help to prevent and cure the COVID-19 infection and periodontitis. 

Codon optimization and codon pair optimization are the strategies to increase gene expression [52]. There are various approaches for codon optimization. Some approaches use the most optimal codons for all instances of a particular amino acid [53], while others adjust codon distribution proportionally to its natural distribution [54]. These approaches incorporate codon harmonization techniques where slow translation regions are identified and maintained to preserve the proper folding of protein to maintain its antigenicity and immunogenicity [55]. Alternatively, rare codons are replaced with abundant ones or codon pairs which are responsible for slow translation are avoided [56].

Therefore, we analyzed the different codon patterns in the present study and the knowledge will help modulate the expression of five genes associated with periodontitis and SARS-CoV-2 pathology. Based on the nucleotide composition of the genome, the codon usage of any organism is decided [57]. Organisms with GC-rich genomes use G/C ending codons, while organisms with AT-rich genomes use A/T ending codons [58]. In our study, *CD147*, *FURIN*, and *TMPRSS2* genes are found to be GC rich and vice versa for *ACE2* and *BMAL1* genes. Based on RSCU, it is evident that G/C ending codons preference in *CD147*, *FURIN*, and *TMPRSS2* gene set, while A/T-ending codons in ACE2 and *BMAL1* and our results are in concordance with the results of human albumin superfamily members, which are AT-rich genes and preference for A/T-ending codons [59].

Rare codons slow down the translation rate due to the rarity of their cognate tRNAs. A linear relationship has been reported between the gene expression level and the number of rare codons. Wang and colleagues [60] engineered a rare codon device to finely tune the expression level of four genes responsible for the fatty acid synthesis II (FASII) pathway in *E. Coli* that not only switches the expression of reporter genes between the On and Off state but also helped in precisely regulating the protein expression at intermediate levels. A proof-of-concept experiment shows that altering the rare codon number might modulate protein expression levels. Similarly, the antibiotic pyoluteorin biosynthetic gene *pltL* encompasses six AGA rare codons, substituting four with CGC and two with CGG significantly enhanced antibiotic production [61].

In the present study CGT (Arg), AGA (Arg), CGA (Arg), TCG (Ser), CTA (Leu), GTA (Val), GGT (Gly) and CCG (Pro) codons were rare in at least four out of five genes. AGA and AGG codons are the rarest codons in *E.coli* [62], and the same is true for *CD147*, *FURIN*, and *TMPRSS2* genes. To attenuate the virus, viral protein production needs to be reduced; therefore, the strategy is to increase the number of rare codons in recoded virus sequences. As a result, particular codons pair with specific codons at a frequency higher than expected, while some codon combinations are avoided. This phenomenon is called codon pair bias. 

Various researchers have used codon pair deoptimization to construct the attenuated virus for vaccine candidate development. Examples are poliovirus [63], avian influenza virus [63], HIV-1 [64], human respiratory syncytial [65], and porcine reproductive and respiratory syndrome virus [66], where segments of viruses have been modified. Similarly, using the information presented here regarding rare codon pairs, the genes *ACE2*, *CD147*, *FURIN*, and *TMPRSS2* may be deoptimized since, apart from participation in pathogenesis, these genes also contribute to physiological roles, so we cannot wholly knock down the genes. Instead, if using the CRISPR-Cas technique if we can recode the genes, the strategy might be used to ameliorate the ailment. Likewise, we will need to enhance the expression of the *BMAL1* gene, which may be carried out using optimized codons in the recoded sequence to be inserted through CRISPR.

In the present study, amongst the genes envisaged the highest utilization of Val (GTG)–initiated codon pairs in *CD147*, *FURIN*, and *TMPRSS2* genes was observed, while in *ACE2* and *BMAL1* genes, Gln (CAA)- and Glu (GAA)-initiated codons were most abundant. On the other hand, in the SARS-CoV-2 isolates, a bias towards valine-initiated (GUU-UUA, GUU-GAA, GUU-UAU, GUU-GUA) and glycine-initiated codon pairs (GGU-GUU, GGU-GAU, GGU-AAA, GGU-GGU) was observed [67]. Consistently there are under- and overrepresentations of codon pairs in all the organisms that appear to influence the translation machinery [68]. The first example of gene expression reduction through codon pair deoptimization was presented by Coleman and colleagues [63]. They generated synthetic poliovirus capsid gene sequences that contained codon pairs specifically rejected in human coding sequences. Such a codon pair deoptimized sequence was inserted in the infectious cDNA clone of poliovirus and a remarkably attenuated viral phenotype owing to the reduced viral protein expression was observed. However, codon pair-deoptimized sequences are predisposed to mistranslation, decreased mRNA stability, and reduced translation efficiency [69]. While attenuating a highly virulent Marek’s disease herpesvirus by codon pair bias deoptimization, the *UL30* gene, encoding for the catalytic subunit of the viral DNA polymerase, was codon pair deoptimized. First, the gene was divided into three segments of equal length, and then the segments were codon pair deoptimized. A minimum of 224 codon pairs deoptimized in one segment resulted in reduced viral plaque size. In comparison, the introduction of changes in 770 codon pairs appeared highly lethal for the virus and no viable progeny was generated [70]. There is no gold standard for the number of deoptimized codon pairs for an effective reduction in protein production. However, some scientists may argue that codon pair deoptimization has such a minuscule effect on gene expression that one needs to introduce hundreds of changes in codon pairs [71]. In the experiment of Coleman and colleagues [63] in poliovirus capsid protein (PV), codon pairs were deoptimized. PV-Min construct contained 631 deoptimized codon pairs, while its segments PV-MinXY and PV-MinZ contained 407 and 224 deoptimized pairs. The F-Luc reporter was used to seeing the translatability of PV’s capsid region. The attenuation order was PV-Min > PV-MinZ > PV-MinXY, corresponding to 631, 224, and 407 deoptimized codon pairs. So, it appears that the reduction in translation is more sequence-specific and less dependent on the number of deoptimized codon pairs.

Contrarily, while deoptimizing HIV, mutant HIV-P minA showed significantly reduced replication only with 15 substitutions [72]. Hence there is broad scope in research related to optimizing the number required for codon pairs to modulate gene expression effectively. The present study provided valuable knowledge regarding the codon usage patterns, codon bias, and information regarding codon context, where, using this knowledge, gene expression patterns may be modulated as per the requirement under the conditions of periodontitis and SARS-CoV-2 infection. After extensively reviewing the literature, we found only five simultaneously upregulated and downregulated genes in both states that were utilized in the study. 

Expression studies have been carried out in mice lungs where IL-1β is transiently expressed using adenoviral gene transfer via intratracheal administration. As a result, the high expression of IL-1β accompanied by a local increase of the pro-inflammatory cytokines IL-6 and TNF-α was observed with evidence of lung injury [73]. The experiment is a proof of concept experiment showing therapeutic interventions using gene transfer techniques. Hydrodynamic gene delivery has been proven effective in a rodent model to transiently express apolipoprotein L-I (*APOL*-I) and haptoglobin-related protein [74]. The gene copies present in human cells can be modified. The proof of concept is the FDA-approved treatment for spinal muscular atrophy (SMA) where the drug Spinraza® modifies the existing SMN2 gene through the intrathecal administration of four loading doses of oligonucleotides in such a way that the *SMN2* gene is produced in higher amounts. Risdiplam® is another SMA therapy where the *SMN2* gene is modified; however, the drug molecule is orally active. Zolgensma® is a more traditional gene therapy where a single intravenous dose is delivered to correct the missing or non-functional copy of the *SMN1* gene [75]. The gendicine gene therapy drug, developed by Shenzhen SiBionoGeneTech and approved by China Food and Drug Administration [76], harbors a copy of the Tp53 gene embedded in the E1 region of human serotype 5 adenovirus (Ad5). Tumor suppressor genes, including *TP53*, *NOTCH1*, *CDKN2A*, *PIK3CA*, and *FBXW7*, are found inactivated in head and neck squamous cell carcinoma (HNSCC). The expression of Tp53 in cancer cells via gene therapy initiates the apoptotic pathways and has antitumor effects.

In the present case, out of the five genes we envisaged, four genes, *ACE2*, *CD147*, *FURIN*, and *TMPRSS2*, which serve as the receptors and co-receptors for SARS-CoV-2 and are overexpressed during COVID-19 infection and in the periodontal tissues of periodontitis patients too, presenting with inflammation, periodontal pathogens, and damage-induced pyroptosis [14], are required to be reduced to an average level. Elevated IL-1β activity is a feature of COVID-19 [77] and periodontitis [78]. *BMAL1*, an immune system regulator, limits the production of the pro-inflammatory cytokine IL-1β [77]. Therefore, the downregulation of *ACE2*, *CD147*, *FURIN*, and *TMPRSS2* genes and the upregulation of *BMAL1* may offer a therapeutic strategy against COVID-19 and periodontitis.

In light of knowledge from gene therapy studies, it is evident now that the desired upregulation or downregulation may be achieved in human tissue using codon usage information. For gene expression reduction, however, many codon changes are required to achieve the desired result, and this is the limitation inherently associated with codon pair deoptimization. The addition of rare codons might help in this context. With that, a uniform strategy cannot be made based on our studies for all the ailments and every disease model; it has to be studied and optimized separately. 

Synonymous mutation influence phenotypic effects, owing to control over mRNA stability and translational regulation. However, our technique has the limitation that it is challenging to pre-judge the changes in which part of the gene will be most effective [79]. 

Presently the gene therapy technique is limited to treating genetic disorders and life-threatening diseases, such as cancer; however, in the future, with the advancement of technology, it will be possible to apply this to other conditions as well, such as in the case of COVID-19 and dental infections, and the codon usage and context analysis of genes may help reach this target.

## 5. Conclusions

We believe that our study about codon usage, codon bias, availability of rare codons, and codon context will help modulate the gene expression pattern within the organism and, therefore, simultaneously, the COVID-19 and periodontitis problem may be addressed. However, more study is required to obtain an optimal number of codon pairs or rare codons to be replaced in order to obtain an optimal level of gene expression, which is required to maintain the normal physiological functions of the *ACE2*, *BMAL1*, *CD147*, *FURIN*, and *TMPRSS2* genes so that the pathological consequences of SARS-CoV-2 infection and periodontitis may be avoided.

## Figures and Tables

**Table 1:** Average RSCU values of *ACE2*, *CD147*, *FURIN*, *TMPRSS2*, and *BMAL1* genes.

CODONS	Single Letter Amino Acid	*ACE2*	*CD147*	*FURIN*	*TMPRSS2*	*BMAL1*
TTT	F	1.104	0.061	0.763	0.545	1.178
TTC		0.896	1.939	1.237	1.455	0.822
TTA	L	0.743	0.000	0.095	0.230	0.965
TTG		0.778	0.136	0.555	0.700	1.233
CTT		1.474	0.136	0.095	0.584	0.778
CTC		0.691	1.683	1.457	1.106	1.205
CTA		0.534	0.349	0.286	0.406	0.730
CTG		1.780	3.697	3.511	2.975	1.090
ATT	I	1.158	0.258	0.607	1.119	1.267
ATC		1.178	2.585	2.355	1.344	0.793
ATA		0.664	0.158	0.037	0.537	0.940
GTT	V	1.474	0.000	0.072	0.243	1.482
GTC		0.637	1.586	1.065	1.356	1.084
GTA		0.473	0.104	0.435	0.209	0.833
GTG		1.416	2.310	2.428	2.192	0.600
TCT	S	1.577	0.712	0.613	1.326	1.790
TCC		1.287	2.294	1.496	1.500	0.837
TCA		1.229	0.712	0.457	1.235	0.814
TCG		0.000	0.475	0.547	0.132	0.182
AGT		0.726	0.298	0.800	0.398	1.241
AGC		1.182	1.507	2.087	1.409	1.136
CCT	P	1.500	0.724	1.154	0.629	0.942
CCC		0.972	2.692	1.608	1.517	0.928
CCA		1.528	0.362	0.683	1.262	1.975
CCG		0.000	0.222	0.555	0.592	0.156
ACT	T	1.162	0.890	0.515	0.790	0.959
ACC		0.826	1.371	1.731	1.579	1.107
ACA		1.804	0.448	0.933	0.948	1.579
ACG		0.208	1.291	0.820	0.683	0.355
GCT	A	1.503	0.648	0.364	1.011	1.143
GCC		0.839	2.536	2.405	1.789	0.511
GCA		1.658	0.260	0.751	0.580	2.228
GCG		0.000	0.555	0.480	0.620	0.119
TAT	Y	1.233	0.000	0.635	0.772	1.362
TAC		0.768	2.000	1.365	1.228	0.638
CAT	H	1.755	0.000	0.421	0.903	0.699
CAC		0.245	2.000	1.579	1.097	1.301
CAA	Q	0.816	0.000	0.230	0.545	0.754
CAG		1.185	2.000	1.770	1.455	1.246
AAT	N	1.412	0.310	0.721	0.571	0.638
AAC		0.589	1.690	1.279	1.429	1.362
AAA	K	1.148	0.222	0.409	0.905	1.236
AAG		0.852	1.778	1.591	1.095	0.764
GAT	D	0.956	0.283	0.597	0.687	0.941
GAC		1.044	1.717	1.403	1.313	1.059
GAA	E	1.360	0.232	0.423	0.767	1.487
GAG		0.640	1.768	1.577	1.233	0.513
TGT	C	1.083	0.000	0.485	0.656	1.157
TGC		0.917	2.000	1.515	1.344	0.843
CGT	R	0.441	0.000	0.375	0.125	0.350
CGC		0.175	2.551	1.186	1.246	0.114
CGA		0.804	0.000	0.507	0.376	1.220
CGG		0.615	1.777	3.174	0.752	1.343
AGA		1.942	0.487	0.080	1.622	0.709
AGG		2.024	1.185	0.678	1.880	2.263
GGT	G	0.436	0.043	0.315	0.356	0.782
GGC		0.568	2.629	2.087	1.164	0.932
GGA		1.738	0.303	0.244	1.015	1.369
GGG		1.258	1.025	1.354	1.464	0.917

**Table 2:** Table presenting codon numbers and normalized codon numbers in transcripts of genes. Normalized codon pair numbers above a value of 5 (five codon pairs per thousand codon pairs) are indicated in bold.

	ACE2 (4249 Codons)			CD147 (1666 Codons)			*FURIN* (5425 Codons)			TMPRSS2 (1522 Codons)			*BMAL1* (30,873 Codons)		
S. No.	Codon Pair	Codon Numbers	Normalized Codon Number	Codon Pair	Codon Numbers	Normalized Codon Number	Codon Pair	Codon Numbers	Normalized Codon Number	Codon Pair	Codon Numbers	Normalized Codon Number	Codon Pair	Codon Numbers	Normalized Codon Number
1	CAA-GAA	22	**5.18**	GUG-CUG	20	**12.0**	GUG-GCC	41	**7.56**	GUG-UGC	9	**5.91**	GAA-UAU	196	**6.35**
2	AAU-GAA	21	4.94	GAG-GAC	18	**10.8**	GCC-AAC	28	**5.16**	CUG-CAG	9	**5.91**	AAA-GAU	165	**5.34**
3	AAA-AAU	20	4.71	GGC-UCC	16	**9.60**	CUG-GGC	28	**5.16**	AAC-CCC	9	**5.91**	GAU-GAA	148	4.79
4	UAU-GAA	19	4.47	CUG-AAG	14	**8.40**	GGC-GAG	27	4.98	AAC-AAU	9	**5.91**	AUA-GAU	148	4.79
5	GAA-AAU	19	4.47	UCA-GAG	12	**7.20**	AAU-GAC	27	4.98	UUG-AAC	6	**5.91**	GAA-AUC	146	4.73
6	CUG-UUC	19	4.47	GUG-AAG	12	**7.20**	GGG-CUG	26	4.79	UUC-AUG	6	**5.91**	AGC-AUG	146	4.73
7	GUU-GGG	18	4.24	GCG-CUG	12	**7.20**	GCC-CCC	26	4.79	UGU-GCC	6	**5.91**	GCA-GAU	130	4.21
8	GAG-AUG	17	4.00	GCC-CUC	12	**7.20**	GAG-GUG	26	4.79	UGG-AUU	6	**5.91**	AUG-GAC	130	4.21
9	GGA-UUC	16	3.77	CUG-GUC	12	**7.20**	CGG-CUG	26	4.79	UGC-AUC	6	**5.91**	AUG-AUU	115	3.72
10	GAA-GAC	16	3.77	CCC-GGC	12	**7.20**	CAG-CAG	26	4.79	UCC-GGG	6	**5.91**	AUG-AAC	110	3.56
11	CAG-AAA	16	3.77	CCC-GAG	12	**7.20**	GUG-GAG	25	4.61	UCC-AAC	6	**5.91**	GAA-UUG	101	3.27
12	AUG-GCA	16	3.77	AUC-AUC	12	**7.20**	CUG-CCC	25	4.61	UAU-GAC	6	**5.91**	UUU-GUC	100	3.24
13	AAA-CCA	16	3.77	ACU-GAC	12	**7.20**	UGG-GCC	21	3.87	UAC-GGG	6	**5.91**	UUG-UUU	100	3.24
14	GAC-CAG	15	3.53	ACG-GCC	12	**7.20**	GGC-UAC	21	3.87	UAC-CAA	6	**5.91**	GUC-UCA	100	3.24
15	GAA-GAG	15	3.53	AAC-GGC	12	**7.20**	GGC-CGG	21	3.87	GUG-UAC	6	**5.91**	GAU-AAA	100	3.24
16	GCA-UAU	14	3.29	UUC-GUG	10	**6.00**	GGC-ACC	21	3.87	GUC-GAU	6	**5.91**	ACA-GAA	100	3.24
17	CUU-GGA	14	3.29	UCC-GAC	10	**6.00**	GAG-GCC	21	3.87	GGG-GCC	6	**5.91**	AAC-UAC	100	3.24
18	AUG-AAU	14	3.29	UCC-AAG	10	**6.00**	GAG-CCC	21	3.87	GGA-UAC	6	**5.91**	GUU-UUA	99	3.21
19	AAA-GCA	14	3.29	GGC-CAG	10	**6.00**	CUG-GCC	21	3.87	GCG-CUG	6	**5.91**	GCA-GCA	99	3.21
20	UUU-CUG	12	2.82	GGC-ACC	10	**6.00**	CUC-ACC	21	3.87	GCC-UGC	6	**5.91**	GCA-AUG	99	3.21
21	UUU-CAA	12	2.82	GCC-GGC	10	**6.00**	CGG-GAC	21	3.87	GCC-GGC	6	**5.91**	GAA-GCA	99	3.21
22	UUG-AAA	12	2.82	GAC-GAC	10	**6.00**	CGG-AAG	21	3.87	GAC-UGG	6	**5.91**	CUA-UCA	99	3.21
23	UUC-CUG	12	2.82	GAC-CAG	10	**6.00**	CAG-GGC	21	3.87	GAC-UCC	6	**5.91**	CCC-UCU	99	3.21
24	UUC-CAU	12	2.82	CUG-GGC	10	**6.00**	CAC-AUC	21	3.87	GAA-AAC	6	**5.91**	CAG-CUC	99	3.21
25	UGU-GAC	12	2.82	AAU-GAC	10	**6.00**	ACC-CUG	21	3.87	GAA-AAA	6	**5.91**	CAA-GGA	99	3.21
26	UGG-AUG	12	2.82	UAC-GAG	8	4.80	AAC-CAC	21	3.87	CUG-AAC	6	**5.91**	AUG-GCU	99	3.21
27	GGG-GAA	12	2.82	GUC-UUC	8	4.80	GCC-UUC	20	3.69	CCU-CUG	6	**5.91**	AGG-AUG	99	3.21
28	GCU-AAU	12	2.82	GUC-CUG	8	4.80	CAG-AAG	20	3.69	CCC-ACU	6	**5.91**	AGG-AUA	99	3.21
29	GAA-GCU	12	2.82	GUC-CGC	8	4.80	CAC-CUG	20	3.69	CAG-UAC	6	**5.91**	ACU-GUU	99	3.21
30	GAA-ACA	12	2.82	GGG-CAG	8	4.80	GUC-UUC	19	3.50	CAG-CCC	6	**5.91**	AAU-GAU	99	3.21

## Data Availability

Available upon request.

## Supplementary Materials

The following supporting information can be downloaded at: https://www.mdpi.com/article/10.3390/vaccines10111874/s1.
